# P76 - Can we successfully collect research data from children with asthma using a SMS system?

**DOI:** 10.1186/2045-7022-4-S1-P131

**Published:** 2014-02-28

**Authors:** Florian Gahleitner, Julian Legg, Emma Holland, Sarah Pearson, Graham Roberts

**Affiliations:** 1 NIHR Respiratory Biomedical Research Unit, University Hospitals of Southampton Foundation Trust, Southampton, United Kingdom

## Introduction

A meaningful analysis in research is only possible with robust, valid data. Paper diaries allow the collection of data from individuals over time but are notorious for poor compliance and reliability. SMS technology is a novel method for data collection in medical research. Time-tagged SMS can be transferred directly to an electronic data file.

## Methods

We used SMS to collect symptoms and peak flow (PEF) meter readings from children with asthma. Digital PEF meters enabled data download to confirm validity of PEF data sent by SMS. Parents responded to 5 SMS messages daily for 7 days during baseline and for 14 days during a cold. Parents provided written feedback about the SMS-based data collection at the beginning (Q1) and at the end (Q2) of the study.

## Results

92% (22/24) and 83% (20/24) of participants completed Q1 and Q2 respectively. All (22/22) were 'very happy' or 'happy' to use SMS for this study. 95% (19/20) found the system user-friendly and 55% (11/20) said that they would more likely participate in a study using SMS for data collection. 25% felt 'sometimes' unhappy about receiving the SMS with 'lack of time' as one problem mentioned. In 3.1% (0, 6.5) SMS remained unanswered, in 6.4% (2.4, 13.7) PEF data sent were not recorded on the meter, in 16.9% (11.5, 23.2) PEF readings sent via SMS did not correlate with the readings stored on the meter. [Numbers represent % Median (25th, 75th percentile)]

## Discussion

This real-time capture of data appears to be well accepted and could avoid some of the pitfalls of backfilled paper diaries although the reliability of data needs further scrutiny.

